# Prevalence of disability among adults with chronic obstructive pulmonary disease, Behavioral Risk Factor Surveillance System 2016–2017

**DOI:** 10.1371/journal.pone.0229404

**Published:** 2020-02-27

**Authors:** Djeneba Audrey Djibo, Jessica Goldstein, Jean G. Ford

**Affiliations:** 1 Division of Research, Department of Medicine, Einstein Healthcare Network, Philadelphia, PA, United States of America; 2 Department of Medicine, Einstein Healthcare Network, Philadelphia, PA, United States of America; Appalachian State University, UNITED STATES

## Abstract

**Background:**

The prevalence of disabilities is rising steadily, reflecting an aging population and an increasing burden of chronic conditions affecting quality of life. There are scant national data on the prevalence of disability among individuals with chronic obstructive pulmonary disease (COPD). The main objective was to estimate the prevalence of common disabilities among US-based individuals diagnosed with COPD.

**Methods:**

Data from the BRFSS, a national telephone survey examining health-related behaviors in 2016–2017 were analyzed. The study population consisted of individuals with self-reported COPD (N = 38352 in 2016 and N = 35423 in 2017). The prevalence of disabilities in hearing, vision, cognition, mobility, and independent living were obtained and adjusted with sampling weights. Healthcare access measures were described by type of disability.

**Results:**

Mobility disability had the highest prevalence of 45.9 (44.8–47.0) % in 2016 and 48.4 (47.3–49.5) % in 2017 among respondents with COPD. The prevalence of disabilities was highest among those 45–64 years old, except for hearing and cognition. Hearing disabilities were most prevalent among males with COPD while cognitive and mobility disabilities were most prevalent among females with COPD. While differences in the prevalence of disabilities were observed, access to health care was similar by disability type and age group among respondents.

**Conclusion:**

Contrary to expectation, the highest prevalence of disabilities was found not to be among those 65 years old and above. Further research is needed to explain this age-specific shift in the burden of disability, as long-term care planning and prevention support systems should be informed by the demographical patterns of disabilities among individuals with COPD.

## Introduction

Chronic obstructive pulmonary disease (COPD) has consistently remained among the top five causes of mortality and morbidity in the United States.[1,2] It is estimated that 15.5 million adults in the United States have received a diagnosis of COPD, corresponding to 5.9% age-adjusted prevalence rate.[1] In 2015, it was estimated that 11.5 per 1000 hospitalizations among Medicare enrollees were due to COPD.[1] Symptoms pertaining to COPD, including chronic bronchitis and emphysema, are typically breathlessness, chronic airway obstruction, and chronic cough. [3] While smoking is the most common risk factor for COPD, exposure to air pollution, repeated respiratory infections, occupational exposure and genetic risk have also been found to be associated with COPD.[1,2,4] There are gender differences in the prevalence of COPD nationally among adults of all ages, as a higher prevalence has been reported among women compared to men, 7.0% vs 4.3% respectively.[2] However, among older adults living in care facilities, COPD prevalence was found to be higher among men compared to women, 14.8% vs 11.4%.[5] Racial disparities exist in the life course of individuals living with COPD. The prevalence of COPD is overall similar between Caucasian and African American adults, but is the lowest among Hispanics/Latinos and Asian American adults.[2] However compared to non-Hispanic whites, African Americans with COPD have a reduced quality of life and an increased risk of mortality.[6]

Individuals with COPD have an increased burden of disease compared to the general population,[6,7] and a wide range of clinical comorbidities including metabolic syndrome, sleep apnea, diabetes, depression, osteoporosis, cardiac failure, ischemic heart disease, pulmonary hypertension, lung cancer, muscle wasting, and cachexia.[8] Comorbidities such as congestive heart failure (CHF), chronic kidney disease (CKD), diabetes, and obstructive sleep apnea (OSA) have been associated to mortality in COPD.[6] However, being diagnosed with COPD is not the only factor influencing the presence of comorbid conditions. Observed risk factors for chronic bronchitis include: smoking, lower education status, underweight individuals, and severely obesity.[4] Being overweight/underweight has been causally related with reduced respiratory function, leading to COPD along with the biological theory of the association between adiposity, endocrine disruption, and inflammation.[9,10] Some of these comorbidities are possibly directly caused by COPD while others such as depression or sleep apnea do not have a characterized direct pathophysiological link with COPD but may nonetheless be related with COPD progression.[8,11]

The prevalence of disability in the adult US population is a public health concern because it can impact quality of life, the individual’s ability to maintain treatment regimens due to complex medical and external burdens, and the adequacy of public health interventions. About one in four adults has a disability, including hearing, vision, cognition, mobility, self-care, and independent living.[12] Overall, mobility was the most prevalent disability type at 13.7%, followed by cognition, 10.8%, and independent living, 6.8%, hearing, 5.9%, vision, 4.6%, and finally self-care, 3.7%.[12] Income, region of residence, quality of accessible health services, and social determinants are all affecting the prevalence of these disabilities.[12,13]

Although functional disabilities are not seen as typical comorbidities associated with COPD, there is limited information on age-specific patterns of disabilities among individuals with COPD. Epidemiological evidence has demonstrated that COPD is associated with impairments in hearing, vision, cognition, mobility, self-care, and the ability to live independently;[14] however there is limited information on the age-specific burden of disabilities among individuals with COPD. This information would be useful, to help those with COPD and disabilities lead the healthiest life possible. The purpose of the present study is to estimate the prevalence of disabilities among adults with COPD, and to examine how the distribution of these disabilities differ by demographic and healthcare access characteristics among individuals with COPD.

## Methods

### Study sample

**Fig 1:**
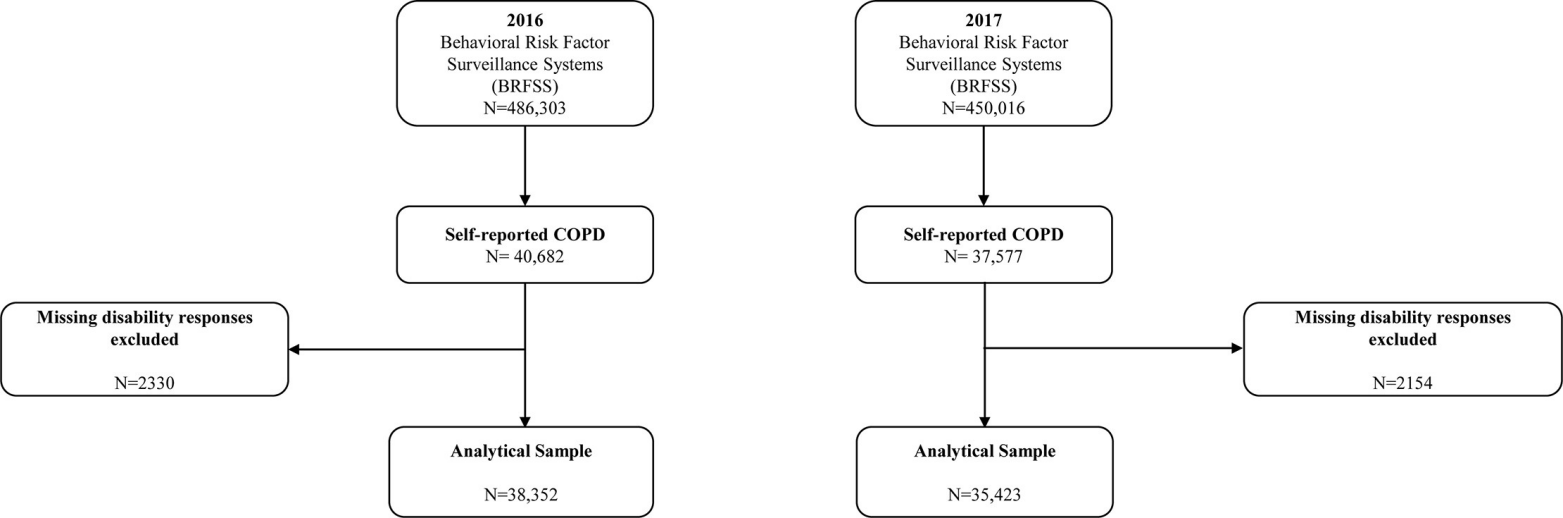
Study sample selection from 2016 and 2017 BRFSS surveys.

The study sample was composed of respondents of the Behavioral Risk Factor Surveillance System (BRFSS) survey, a national telephone survey of US dwellers encompassing various chronic conditions, general health status and health behaviors.[15] Participants are contacted through random digit dialing, and only adults 18 years and older are eligible to answer the questionnaire. Eligible participants were reached via either landline or mobile phone. Median response rates among those reached over landlines in 2016 and 2017 were 47.7% and, 45.3%, respectively; and via cell phone, 46.4%and 44.5%, respectively.[16] There were 486,303 respondents in 2016 and 450,016 respondents in 2017. The sample was restricted to those who provided information regarding their COPD status and about disabilities, 38,352 (8.4%) in 2016 and 35,423 (8.4%) in 2017 respectively (see Fig 1). A COPD case was defined as responding positively to being “ever told that they have COPD, emphysema, or chronic bronchitis”.

### Study measures

The national survey included questions about disability, specifically concerning hearing (“are you deaf or do you have difficulty hearing?”), vision (“are you blind or do you have serious difficulty seeing, even when wearing glasses?”), cognition (“because of a physical, mental, or emotional condition, do you have serious difficulty concentrating?”), self-care (“do you have difficulty dressing or bathing?”), mobility (“do you have serious difficulty walking or climbing stairs?”), and independent living, (“because of a physical, mental or emotional condition, do you have difficulty doing errands alone such as visiting a doctor’s office or shopping?”). Those responding “yes” were classified as having the disability. Missing responses (5.7% of sample for each survey year) were excluded from the analysis, n = 2330 in 2016, n = 2154 in the 2017 BRFSS. In addition, the prevalence of any of the 6 disabilities was dichotomized based on answering “yes” to at least one disability, or “no” to all disability questions. The questionnaire also collected information on age group, mainly 18–44, 45–64 and 65 years old and above, sex (male or female), race/ethnicity, categorized as Caucasian, African-American, Hispanic, American Indian/Alaska Native, multiracial and other race/ethnicity responses, and finally US Census regions (Northeast, Midwest, South, and West). Four measures of healthcare access were also analyzed by disability type and age group among respondents with COPD: insurance, primary care provider, yearly health check-up and economic barriers to meeting health care needs.

### Statistical analysis

Yearly prevalence estimates were obtained for 2016 and 2017 surveys separately. Adjusted prevalence of the six disabilities of interest and of any disability was calculated, using sampling weight corrections. Similarly, the adjusted rates of healthcare access were obtained. All analyses were stratified by age groups, 18–44, 45–64, and 65 and above. Estimates with 95% confidence intervals were computed using SAS v.9.4 (Cary, NC). IRB approval was not obtained as the data used here are from publicly available deidentified national surveillance system.

## Results

**Fig 2:**
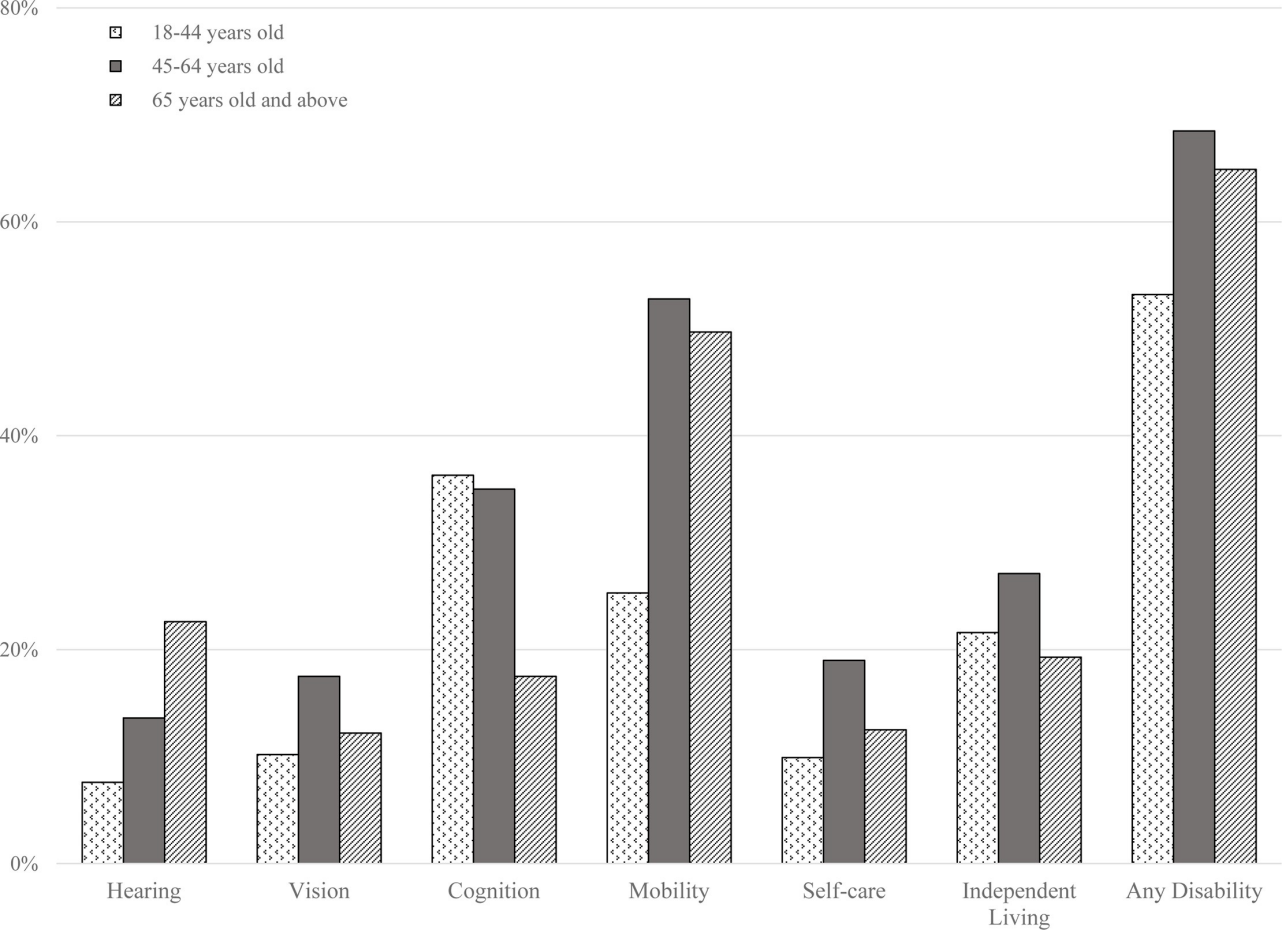
Weighted unadjusted prevalence estimates by type of disability by age-group among persons with self-reported COPD, BRFSS 2016.

Weighted prevalence estimates of demographic characteristics and self-reported disabilities are presented in Table 1 for 2016 and Table 2 for 2017, respectively. Adjusting for survey sampling, 41.4% of respondents with self-reported COPD were 45–64 years old (95%CI: 40.4–42.5%), 57.2% were female (95%CI: 56.2–58.3%), and most (72%) were Caucasian (95%CI: 71.7–73.8%) in 2016. The largest proportion (41.7%, 95%CI: 41.1–42.3%) were residents of the South of the United States. Among survey respondents with self-reported COPD, 15.7% had a hearing disability (95%CI: 15.0–16.5%), 14.0% a vision disability (95%CI: 13.2–14.7%), 28.7% a cognitive disability (95%CI: 27.7–29.7%), 45.9% mobility issues (95%CI: 44.8–47.0), 14.7% a self-care disability (95%CI: 13.9–15.5%), and 23.0% had difficulty doing independent errands (95%CI: 22.1–23.9%). Moreover, 64.0% had at least one functional disability in addition to having COPD (95%CI: 62.9–65.0%). Among 18-44-year-old group, the most prevalent disability was cognitive impairment (36.5%; 95% CI: 33.9–39.1%), and the least prevalent was hearing impairment (7.6%. 95%CI: 6.3–8.8%), see Fig 2. Among the middle-age group, mobility problems were most prevalent disability (52.8%; 95%CI: 51.1–54.4%), and hearing disability was the least prevalent (13.6%; 95%CI: 12.6–14.7%). Finally, among those in the age group 65 and above age group, mobility problems were the most common disability, (49.7%; 95%CI: 48.2–51.2%) and vision problem were the least common (12.2%; 95%CI: 11.2–13.1%). The prevalence of any disabilities was generally highest among female respondents in 2016. Females with COPD surveyed were found with higher rates of cognition 30.5% (95%CI: 29.3–31.8%), mobility 48.5% (95%CI: 47.1–49.8%), and independent living 26.2% (95%CI: 25.0–27.4%), disabilities (Table 1). The prevalence of hearing disabilities was only higher among males with COPD surveyed 20.4% (95%CI: 19.0–21.8%). Disparities in the prevalence of disabilities by race/ethnicity were also found. American Indian / Alaska Native respondents with COPD had the highest rate of any disability, 82.2% (95%CI: 79.9–84.6%), and reported the highest rates of hearing 24.5% (95%CI: 21.6–27.4%), cognition 45.4% (95%CI: 41.9–49.0%), and mobility 60.4% (95%CI: 57.0–63.7%), disabilities.

**Table 1:** Weighted unadjusted prevalence estimates by type of disability among persons with self-reported COPD, BRFSS 2016.

	Total		Hearing	Vision	Cognition	Mobility	Self-care	Independent Living	Any Disability
	N	% (95% CI)	% (95% CI)	% (95% CI)	% (95% CI)	% (95% CI)	% (95% CI)	% (95% CI)	% (95% CI)
Total	38352		15.7 (15.0–16.5)	14.0 (13.2–14.7)	28.7 (27.7–29.7)	45.9 (44.8–47.0)	14.7 (13.9–15.5)	23.0 (22.1–23.9)	64.0 (62.9–65.0)
Sex									
Male	14408	42.7 (41.7–43.8)	20.4 (19.0–21.8)	12.8 (11.7–14.0)	26.2 (24.6–27.9)	42.5 (40.8–44.1)	14.1 (12.8–15.4)	18.8 (17.3–20.2)	62.1 (60.5–63.8)
Female	23939	57.2 (56.2–58.3)	12.3 (11.5–13.1)	14.9 (13.9–15.8)	30.5 (29.3–31.8)	48.5 (47.1–49.8)	15.1 (14.2–16.0)	26.2 (25.0–27.4)	65.3 (64.0–66.7)
*Missing*	*5*								
Race/Ethnicity									
White	30680	72.7 (71.7–73.8)	17.0 (16.2–17.8)	12.0 (11.3–12.7)	26.5 (25.5–27.5)	45.9 (44.8–47.0)	13.2 (12.4–13.9)	21.4 (20.6–22.3)	63.5 (62.4–64.5)
Black	2897	12.0 (11.2–12.8)	9.2 (7.5–10.9)	19.6 (16.5–22.8)	30.2 (27.0–33.4)	48.1 (44.7–51.4)	18.0 (15.6–20.4)	24.2 (21.4–27.1)	64.5 (60.9–68.0)
Hispanic	1773	9.2 (8.5–9.9)	10.6 (8.7–12.5)	21.1 (17.9–24.3)	33.6 (29.9–37.3)	41.6 (37.8–45.4)	18.0 (15.0–21.0)	27.1 (23.5–30.7)	63.6 (59.9–67.4)
AI/AN	798	1.5 (1.3–1.7)	24.5 (21.6–27.4)	17.4 (14.5–20.3)	45.4 (41.9–49.0)	60.4 (57.0–63.7)	22.8 (18.8–26.7)	37.1 (33.9–40.3)	82.2 (79.9–84.6)
Multiracial	1111	2.5 (2.0–2.9)	17.1 (13.4–20.7)	16.3 (13.0–19.7)	46.6 (41.5–51.7)	55.8 (51.2–60.5)	28.3 (24.4–32.2)	41.3 (36.2–46.3)	76.7 (72.8–80.7)
Other	456	2.1 (1.5–2.7)	20.8 (4.9–36.6)	5.1 (2.4–7.8)	34.6 (18.5–50.7)	24.8 (15.7–34.0)	5.1 (2.9–7.4)	4.7 (8.3–26.6)	49.6 (34.2–64.9)
*Missing*	*637*								
US Census Region									
Northeast	7438	16.7 (16.2–17.2)	12.7 (11.0–14.4)	12.3 (10.4–14.2)	25.9 (23.5–28.2)	44.1 (41.5–46.7)	12.0 (10.4–13.6)	19.9 (17.9–21.9)	60.2 (57.7–62.8)
Midwest	8721	23.3 (22.8–23.7)	16.7 (15.3–18.1)	12.8 (11.5–14.1)	27.8 (26.1–29.6)	44.5 (42.6–46.4)	14.2 (12.8–15.6)	21.9 (20.4–23.4)	64.1 (62.3–66.0)
South	15046	41.7 (41.1–42.3)	16.1 (15.0–17.2)	16.1 (14.8–17.4)	31.2 (29.6–32.9)	49.8 (48.1–51.5)	16.4 (15.0–17.7)	25.9 (24.3–27.6)	67.0 (65.4–68.7)
West	6735	18.3 (17.8–18.9)	16.5 (14.1–18.9)	11.3 (9.8–12.8)	26.8 (24.2–29.5)	40.4 (37.8–43.0)	13.5 (11.8–15.3)	20.5 (18.5–22.6)	60.0 (57.1–62.8)
*Missing*	*412*								

AI: American Indian, AN: Alaska Native

**Table 2:** Weighted unadjusted prevalence estimates by type of disability among persons with self-reported COPD, BRFSS 2017.

	Total		Hearing	Vision	Cognition	Mobility	Self-care	Independent Living	Any Disability
	N	% (95% CI)	% (95% CI)	% (95% CI)	% (95% CI)	% (95% CI)	% (95% CI)	% (95% CI)	% (95% CI)
Total	35423		16.8 (15.9–17.6)	14.9 (14.0–15.8)	28.9 (27.8–29.9)	48.4 (47.3–49.5)	16.6 (15.8–17.5)	24.7 (23.7–25.7)	65.6 (64.6–66.7)
Sex									
Male	13735	42.6 (41.5–43.7)	20.8 (19.5–22.2)	13.9 (12.6–15.3)	25.5 (23.9–27.0)	43.1 (41.5–44.8)	15.3 (14.1–16.5)	19.7 (18.3–21.2)	62.8 (61.1–64.4)
Female	21673	57.4 (56.3–58.5)	13.8 (12.7–14.9)	15.6 (14.5–16.7)	31.4 (30.1–32.8)	52.3 (50.8–53.7)	17.6 (16.4–18.7)	28.4 (27.1–29.6)	67.7 (66.4–69.0)
*Missing*	*15*								
Race/Ethnicity									
White	28052	74.5 (73.5–75.5)	17.3 (16.4–18.2)	12.7 (11.9–13.6)	26.6 (25.5–27.7)	47.3 (46.1–48.5)	15.0 (14.2–15.8)	23.3 (22.3–24.4)	64.5 (63.3–65.6)
Black	2787	11.9 (11.1–12.7)	12.4 (9.1–15.7)	21.6 (18.0–25.1)	32.7 (28.9–36.5)	53.1 (49.4–56.8)	22.9 (19.7–26.0)	29.0 (25.7–32.3)	66.8 (63.5–70.1)
Hispanic	1510	8.2 (7.5–8.8)	15.2 (12.1–18.3)	20.9 (17.7–24.1)	35.4 (31.2–39.7)	46.8 (42.6–50.9)	20.4 (17.1–23.7)	26.3 (22.9–29.6)	68.6 (64.5–72.7)
AI/AN	903	1.8 (1.5–2.0)	19.6 (16.4–22.8)	22.5 (20.1–24.8)	43.5 (39.7–47.2)	60.2 (57.0–63.3)	23.2 (19.8–26.5)	34.7 (31.4–38.0)	79.8 (76.7–82.8)
Multiracial	1017	2.0 (1.7–2.2)	19.6 (16.7–22.5)	18.5 (15.3–21.7)	37.3 (33.7–40.8)	51.6 (47.9–55.2)	18.1 (15.7–20.4)	30.9 (27.7–34.0)	71.7 (68.5–74.9)
Other	451	1.7 (1.3–2.1)	23.6 (18.2–30.0)	12.8 (10.9–14.7)	39.5 (29.4–49.5)	49.1 (39.0–59.2)	15.9 (10.2–21.6)	26.2 (19.5–33.0)	61.7 (51.3–72.2)
*Missing*	*703*								
US Census Region									
Northeast	5596	16.0 (15.6–16.4)	15.0 (13.2–16.8)	12.3 (10.7–14.0)	26.8 (24.5–29.2)	44.8 (42.3–47.4)	15.6 (13.7–17.5)	23.6 (21.3–25.9)	62.9 (60.4–65.3)
Midwest	10117	23.2 (22.8–23.7)	15.7 (14.5–16.8)	13.5 (12.2–14.8)	28.5 (26.9–30.1)	50.2 (48.5–52.0)	17.2 (15.7–18.7)	25.2 (23.6–26.8)	66.8 (65.2–68.5)
South	13205	43.0 (42.4–43.7)	17.8 (16.3–19.3)	16.7 (15.1–18.3)	30.4 (28.7–32.2)	50.3 (48.4–52.1)	17.7 (16.2–19.1)	25.7 (24.1–27.3)	67.1 (65.3–68.8)
West	6210	17.7 (17.2–18.3)	17.4 (14.9–19.8)	13.4 (11.1–15.7)	27.4 (24.4–30.4)	44.1 (41.1–47.1)	14.0 (11.9–16.2)	22.1 (19.5–24.6)	62.3 (59.4–65.3)
*Missing*	*295*								

AI: American Indian, AN: Alaska Native

**Fig 3:**
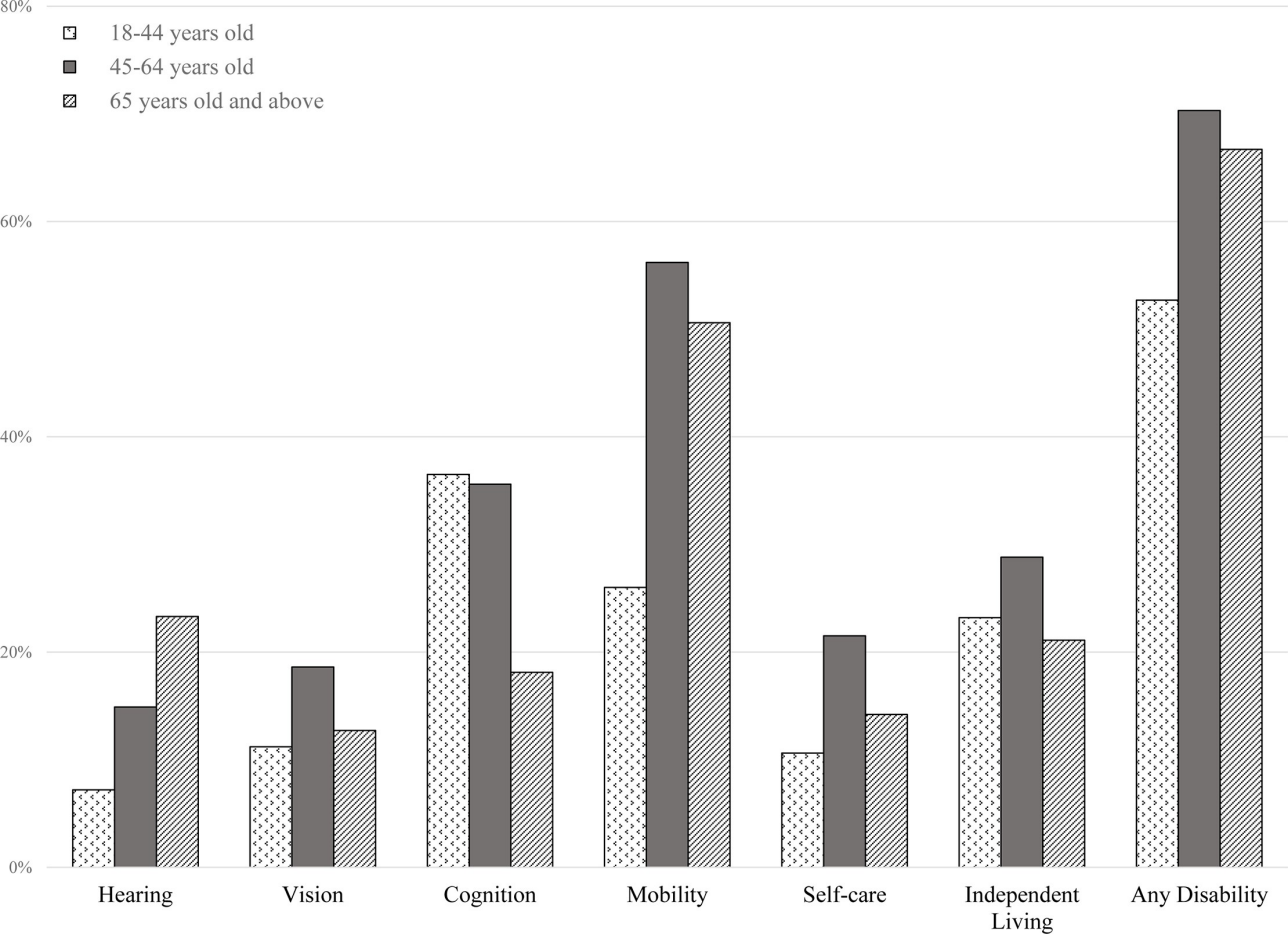
Weighted unadjusted prevalence estimates by type of disability by age-group among persons with self-reported COPD, BRFSS 2017.

Weighted prevalence estimates of demographic characteristics and self-reported disabilities are presented in Table 2 for 2017. Similarly adjusting for survey sampling, 42.1% of survey respondents with self-reported COPD were 45–64 years old (95%CI:41.0–43.2%), 57.4% were female (95%CI: 56.3–58.5%), and mostly Caucasian (74.5%, 95%CI: 73.5–75.5%) in 2017. The largest proportion, 43.0%, 95%CI: 42.4–43.7%, were resident of the South region of the United States. Moreover, 16.8% had a hearing disability (95%CI: 15.9–17.6%), 14.9% a vision disability (95%CI: 95%CI: 14.0–15.8%), 28.9% a cognition disability (95%CI: 27.8–29.9%), 48.4% mobility issues (95%CI: 47.3–49.5), 16.6% a self-care disability (95%CI: 15.8–17.5%), and 24.7% had difficulty doing independent errands (95%CI: 23.7–25.7%). In addition, 65.6% had at least one functional disability in addition to having COPD (95%CI: 64.6–66.7%). Among those 18–44 years old, the highest prevalence of disability concerned cognition, 36.5% (95% CI: 33.9–39.1%) and the lowest prevalence was about impaired hearing, 7.2% (95%CI:5.9–8.5%), see Fig 3. Among those 45–64 years old, the highest rate of disability was about mobility issues, 56.2% (95%CI: 54.5–57.9%), and the lowest rate for hearing disability, 14.9% (95%CI: 13.7–16.1%). Finally, among those in the 65 and above age group, the highest rate of disability was mobility 50.6% (95%CI: 49.0–52.1%) and the lowest rate concerned vision issues, 12.7% (95%CI: 11.5–13.9%). The prevalence of any disabilities was highest among female respondents in 2017. Females with COPD surveyed were found with higher rates of cognition 31.4% (95%CI: 30.1–32.8%), mobility, and independent living disabilities (Table 2). The prevalence of hearing disabilities was only higher among males with COPD surveyed 20.8% (95%CI: 19.5–22.2%). Disparities in the prevalence of disabilities by race/ethnicity were also found. American Indian/Alaska Native respondents with COPD had the highest rate of any disability, 79.8% (95%CI: 76.7–82.8%), and reported the highest rates of cognition 43.1% (95%CI: 41.5–44.8%), and mobility 60.2% (95%CI: 57.0–63.3%), disabilities. Moreover, African-Americans with COPD consistently reported higher rates of disability than Caucasians except for the prevalence of hearing disability.

Regarding measures of healthcare access among respondents with COPD, we found consistently high rates of insurance coverage, across the types of disability in 2016 and 2017, shown in Tables 3–6. Overall, 90.9% had health insurance coverage in 2016 (95%CI: 90.2–91.5%), and 90.5% in 2017(95%CI: 89.7–91.3%), see Table 3. The rate of health insurance coverage was also consistently high among all age groups with COPD, although the 18–44 years old had the lowest rate of the health insurance coverage (Table 4). Individuals 18–44 years old with reported visual or hearing disabilities also had the lowest rates of health insurance coverage 2016 and in 2017: 74.8% (95%CI: 69.8–79.7%) and 75.2% (95%CI 70.6–79.8%), respectively. Moreover, in 2016, most respondents with at least one disability and COPD, also had a usual care provider, with rates ranging from 70.8% (95%CI: 67.5–74.0%) among the 18–44 year-old group to 98.2% (95%CI: 95.8–96.9%) among those 65 years and above. In 2017 coverage rates among those with at least one disability and COPD ranged from 75.6% (95%CI: 73.0–78.2%) in the youngest age group to 95.0% (95%CI: 94.0–96.0%) in the oldest surveyed. Among the 45–64 age group, the rates of cost issues hindering health care needs were 33.3% (95%CI: 30.6–35.9%) and 29.1% (95%CI: 27.2–31.1%) in 2016 and 2017, respectively, and highest among those reporting a visual disability for both years (Table 5). The rates of a recent routine check-up among all subjects with COPD were 82.1% (95%CI 81.0–83.1%) and 82.4% (95%CI: 81.2–83.5%) in 2016 and 2017, respectively. Routine medical check-up rates in the last year were lowest among the youngest group and highest among those 65 and older.

**Table 3:** Weighted unadjusted prevalence of health care access by type of disability among those with self-reported COPD, BRFSS 2016–2017.

		All		Hearing	Vision	Cognition	Mobility	Self-care	Independent Living	Any Disability
		N	% (95% CI)	% (95% CI)	% (95% CI)	% (95% CI)	% (95% CI)	% (95% CI)	% (95% CI)	% (95% CI)
**2016**										
Health insurance coverage										
	Yes	36055	90.9 (90.2–91.5)	91.7 (90.3–93.2)	87.5 (85.8–89.3)	88.6 (87.3–90.0)	92.9 (92.1–93.7)	92.8 (91.5–94.1)	90.5 (89.0–91.9)	91.1 (90.3–91.9)
	No	2182	9.1 (8.5–9.8)	8.3 (6.8–9.7)	12.5 (10.7–14.2)	11.4 (10.0–12.7)	7.1 (6.3–7.9)	7.2 (5.9–8.5)	9.5 (8.1–11.0)	8.9 (8.1–9.7)
	*Missing*	*115*								
Usual health care provider										
	Yes	35092	87.9 (87.2–88.7)	90.6 (89.1–92.0)	86.3 (84.0–88.6)	85.9 (84.4–87.4)	91.6 (90.7–92.6)	92.1 (90.9–93.4)	89.9 (88.6–91.2)	89.0 (88.1–89.9)
	No	3130	12.1 (11.3–12.8)	9.4 (8.0–10.9)	13.7 (11.4–16.0)	14.1 (12.6–15.6)	8.4 (7.4–9.3)	7.9 (6.6–9.1)	10.1 (8.8–11.4)	11.0 (10.1–11.9)
	*Missing*	*130*								
Unmet health care need because of cost during past 12 months										
	Yes	6264	20.6 (19.6–21.5)	20.3 (18.6–22.0)	33.3 (30.6–35.9)	32.6 (30.6–34.7)	23.6 (22.2–25.0)	30.3 (27.3–33.2)	31.0 (28.7–33.3)	24.4 (23.1–25.6)
	No	31979	79.4 (78.5–80.4)	79.7 (78.0–81.4)	66.7 (64.1–69.4)	67.4 (65.3–69.4)	76.4 (75.0–77.8)	69.7 (66.8–72.7)	69.0 (66.7–71.3)	75.6 (74.4–76.9)
	*Missing*	*109*								
Routine check-up during past 12 months										
	Yes	31841	80.6 (79.7–81.5)	83.9 (82.1–85.7)	79.0 (76.5–81.4)	78.1 (76.3–79.9)	84.8 (83.6–85.9)	84.0 (82.2–85.8)	82.7 (81.1–84.4)	82.1 (81.0–83.1)
	No	5956	19.4 (18.5–20.3)	16.1 (14.3–17.9)	21.0 (18.6–23.5)	21.9 (20.1–23.7)	15.2 (14.1–16.4)	16.0 (14.2–17.8)	17.3 (15.6–18.9)	17.9 (16.9–19.0)
	*Missing*	*555*								
**2017**										
Health insurance coverage										
	Yes	33145	90.5 (89.7–91.3)	92.4 (90.2–94.5)	88.5 (86.2–90.8)	88.5 (86.9–90.1)	92.4 (91.3–93.5)	91.2 (89.1–93.3)	90.9 (89.5–92.2)	90.7 (89.7–91.7)
	No	2183	9.5 (8.7–10.3)	7.6 (5.5–9.8)	11.5 (9.2–13.8)	11.5 (9.9–13.1)	7.6 (6.5–8.7)	8.8 (6.7–10.9)	9.1 (7.8–10.5)	9.3 (8.3–10.3)
	*Missing*	*95*								
Usual health care provider										
	Yes	32232	87.6 (86.7–88.4)	92.0 (90.8–93.3)	88.3 (86.3–90.3)	87.1 (85.8–88.4)	91.9 (90.9–92.9)	92.1 (90.8–93.4)	90.6 (89.3–91.9)	89.7 (88.7–90.6)
	No	3082	12.4 (11.6–13.3)	8.0 (6.7–9.2)	11.7 (9.7–13.7)	12.9 (11.6–14.2)	8.1 (7.1–9.1)	7.9 (6.6–9.2)	9.4 (8.1–10.7)	10.3 (9.3–11.3)
	*Missing*	*109*								
Unmet health care need because of cost during past 12 months										
	Yes	5937	21.0 (20.0–22.0)	21.6 (19.4–23.7)	32.2 (29.8–34.5)	31.8 (29.9–33.7)	23.2 (21.7–24.7)	28.8 (26.5–31.2)	27.7 (25.8–29.5)	23.8 (22.6–25.1)
	No	29369	79.0 (78.0–80.0)	78.4 (76.3–80.6)	67.8 (65.5–70.2)	68.2 (66.3–70.1)	76.8 (75.3–78.3)	71.2 (68.8–73.5)	72.3 (70.5–74.2)	76.2 (74.9–77.4)
	*Missing*	*117*								
Routine check-up during past 12 months										
	Yes	29141	79.9 (78.9–80.9)	85.4 (83.3–87.4)	80.6 (78.6–82.7)	78.3 (76.4–80.1)	84.7 (83.4–86.0)	83.3 (80.9–85.6)	82.5 (80.7–84.4)	82.4 (81.2–83.5)
	No	5786	20.1 (19.1–21.1)	14.6 (12.6–16.7)	19.4 (17.3–21.4)	21.7 (19.9–23.6)	15.3 (14.0–16.6)	16.7 (14.4–19.1)	17.5 (15.6–19.3)	17.6 (16.5–18.8)
	*Missing*	*496*								

**Table 4:** Weighted unadjusted prevalence of health care access by type of disability among 18–44 years old with self-reported COPD, BRFSS 2016–2017.

		All		Hearing	Vision	Cognition	Mobility	Self-care	Independent Living	Any Disability
		N	% (95% CI)	% (95% CI)	% (95% CI)	% (95% CI)	% (95% CI)	% (95% CI)	% (95% CI)	% (95% CI)
**2016**										
Health insurance coverage										
	Yes	3037	79.6 (77.3–81.8)	76.3 (69.0–83.5)	74.8 (69.8–79.7)	78.9 (75.8–81.9)	84.7 (81.5–87.9)	85.9 (82.6–89.2)	78.9 (74.3–83.6)	79.4 (76.4–82.4)
	No	623	20.4 (18.2–22.7)	23.7 (16.5–31.0)	25.2 (20.3–30.2)	21.1 (18.1–24.2)	15.3 (12.1–18.5)	14.1 (10.8–17.4)	21.1 (16.4–25.7)	20.6 (17.6–23.6)
	*Missing*	29								
Usual health care provider										
	Yes	2698	69.8 (67.4–72.3)	73.4 (66.3–80.5)	70.1 (65.4–74.8)	72.0 (68.7–75.2)	75.8 (71.7–79.9)	80.8 (76.5–85.1)	75.2 (71.1–79.3)	70.8 (67.5–74.0)
	No	968	30.2 (27.-32.6)	26.6 (19.5–33.7)	29.9 (25.2–34.6)	28.0 (24.8–31.3)	24.2 (20.1–28.3)	19.2 (14.9–23.5)	24.8 (20.7–28.9)	29.2 (26.0–32.5)
	*Missing*	23								
Unmet health care need because of cost during past 12 months										
	Yes	1238	33.4 (30.9–35.9)	39.3 (32.6–46.0)	50.5 (45.9–55.0)	43.7 (40.3–47.2)	40.5 (36.0–45.0)	47.4 (41.3–53.5)	49.3 (44.5–54.1)	40.9 (37.4–44.5)
	No	2438	66.6 (64.1–69.1)	60.7 (54.0–67.4)	49.5 (45.0–54.1)	56.3 (52.8–59.7)	59.5 (55.0–64.0)	52.6 (46.5–58.7)	50.7 (45.9–55.5)	59.1 (55.5–62.6)
	*Missing*	13								
Routine check-up during past 12 months										
	Yes	2334	62.3 (59.7–64.8)	64.7 (57.3–72.1)	57.8 (53.6–61.9)	63.0 (59.3–66.7)	71.4 (67.4–75.3)	72.3 (67.0–77.7)	68.2 (63.5–72.9)	64.3 (61.0–67.7)
	No	1282	37.7 (35.2–40.3)	35.3 (27.9–42.7)	42.2 (38.1–46.4)	37.0 (33.3–40.7)	28.6 (24.7–32.6)	27.7 (22.3–33.0)	31.8 (27.1–36.5)	35.7 (32.3–39.0)
	*Missing*	73								
**2017**										
Health insurance coverage										
	Yes	2665	79.4 (77.3–81.5)	75.2 (70.6–79.8)	78.8 (74.6–83.0)	80.2 (77.3–83.1)	86.2 (83.5–88.9)	89.1 (86.3–92.0)	87.2 (84.1–90.3)	79.5 (76.8–82.1)
	No	612	20.6 (18.5–22.7)	24.8 (20.2–29.4)	21.2 (17.0–25.4)	19.8 (16.9–22.7)	13.8 (11.1–16.5)	10.9 (8.0–13.7)	12.8 (9.7–15.9)	20.5 (17.9–23.2)
	*Missing*	21								
Usual health care provider										
	Yes	2360	68.2 (65.4–70.9)	77.1 (71.5–82.7)	81.4 (77.9–84.9)	74.7 (71.8–77.7)	83.7 (81.5–86.0)	85.8 (83.7–87.8)	80.5 (77.3–83.6)	75.6 (73.0–78.2)
	No	923	31.8 (29.1–34.6)	22.9 (17.3–28.5)	18.6 (15.1–22.1)	25.3 (22.3–28.2)	16.3 (14.0–18.5)	14.2 (12.2–16.3)	19.5 (16.4–22.7)	24.4 (21.8–27.0)
	*Missing*	15								
Unmet health care need because of cost during past 12 months										
	Yes	1136	36.5 (33.5–39.5)	48.7 (44.0–53.4)	51.9 (47.2–56.6)	42.9 (39.2–46.7)	40.9 (37.1–44.8)	40.7 (35.1–46.4)	37.6 (33.6–41.5)	42.6 (39.5–45.8)
	No	2149	63.5 (60.5–66.5)	51.3 (46.6–56.0)	48.1 (43.4–52.8)	57.1 (53.3–60.8)	59.1 (55.2–62.9)	59.3 (53.6–64.9)	62.4 (58.5–66.4)	57.4 (54.2–60.5)
	*Missing*	13								
Routine check-up during past 12 months										
	Yes	2009	59.8 (56.8–62.9)	63.8 (58.9–68.6)	68.0 (63.1–72.8)	63.2 (59.5–66.9)	74.5 (71.3–77.7)	77.8 (72.7–82.3)	71.3 (67.6–75.0)	65.3 (62.2–68.3)
	No	1220	40.2 (37.1–43.2)	36.2 (31.4–41.1)	32.0 (27.2–36.9)	36.8 (33.1–40.5)	25.5 (22.3–28.7)	22.5 (17.7–27.3)	28.7 (25.0–32.4)	34.7 (31.7–37.8)
	*Missing*	69								

**Table 5:** Weighted unadjusted prevalence of health care access by type of disability among 45–64 years old with self-reported COPD, BRFSS 2016–2017.

		All		Hearing	Vision	Cognition	Mobility	Self-care	Independent Living	Any Disability
		N	% (95% CI)	% (95% CI)	% (95% CI)	% (95% CI)	% (95% CI)	% (95% CI)	% (95% CI)	% (95% CI)
**2016**										
Health insurance coverage										
	Yes	13558	89.7 (88.7–90.7)	87.4 (84.9–89.8)	85.3 (82.8–87.8)	89.5 (87.8–91.2)	90.2 (88.9–91.6)	92.0 (90.4–93.6)	90.6 (88.7–92.6)	89.5 (88.3–90.8)
	No	1294	10.3 (9.3–11.3)	12.6 (10.2–15.1)	14.7 (12.2–17.2)	10.5 (8.8–12.2)	9.8 (8.4–11.1)	8.0 (6.4–9.6)	9.4 (7.4–11.3)	10.5 (9.2–11.7)
	*Missing*	42								
Usual health care provider										
	Yes	13488	89.3 (88.3–90.4)	87.0 (54.6–89.3)	85.6 (82.1–89.0)	89.3 (87.5–91.2)	91.1 (89.7–92.5)	92.7 (91.1–94.3)	91.5 (90.0–93.1)	89.6 (88.3–90.9)
	No	1369	10.7 (9.6–11.7)	13.0 (10.7–15.4)	14.4 (11.0–17.9)	10.7 (8.8–12.5)	8.9 (7.5–10.3)	7.3 (5.7–8.9)	8.5 (6.9–10.0)	10.4 (9.1–11.7)
	*Missing*	37								
Unmet health care need because of cost during past 12 months										
	Yes	3419	25.4 (23.8–27.0)	31.0 (28.0–34.0)	38.4 (34.8–42.1)	33.9 (31.3–36.5)	29.9 (27.7–32.1)	34.7 (30.2–39.1)	34.2 (30.7–37.7)	29.9 (27.9–31.9)
	No	11432	74.6 (73.0–76.2)	69.0 (66.0–72.0)	61.6 (57.9–65.2)	66.1 (63.5–68.7)	70.1 (67.9–72.3)	65.3 (60.9–69.8)	65.8 (62.3–69.3)	70.1 (68.1–72.1)
	*Missing*	43								
Routine check-up during past 12 months										
	Yes	11903	80.3 (78.9–81.6)	77.7 (74.9–80.6)	77.8 (74.1–81.5)	80.8 (78.3–83.2)	82.5 (80.6–84.4)	83.8 (81.4–86.2)	83.2 (81.1–85.4)	81.1 (79.5–82.7)
	No	2789	19.7 (18.4–21.1)	22.3 (19.4–25.1)	22.2 (18.5–25.9)	19.2 (16.8–21.7)	17.5 (15.6–19.4)	16.2 (13.8–18.6)	16.8 (14.6–18.9)	18.9 (17.3–20.5)
	*Missing*	202								
**2017**										
Health insurance coverage										
	Yes	12378	88.8 (87.6–90.0)	89.0 (85.9–92.2)	87.0 (84.4–89.6)	89.5 (87.7–91.4)	90.1 (88.6–91.5)	90.8 (88.7–92.9)	90.8 (89.2–92.3)	89.1 (87.7–90.5)
	No	1305	11.2 (10.0–12.4)	11.0 (7.8–14.1)	13.0 (10.4–15.6)	10.5 (8.6–12.3)	9.9 (8.5–11.4)	9.2 (7.1–11.3)	9.2 (7.7–10.8)	10.9 (9.5–12.3)
	*Missing*	40								
Usual health care provider										
	Yes	12357	88.7 (87.4–90.0)	88.8 (86.1–91.4)	87.5 (85.4–89.5)	89.6 (88.0–91.1)	90.9 (89.1–92.6)	92.0 (90.5–93.5)	92.3 (91.0–93.6)	89.6 (88.1–91.1)
	No	1328	11.3 (10.0–12.6)	11.2 (8.6–13.9)	12.5 (10.5–14.6)	10.4 (8.9–12.0)	9.1 (7.4–10.9)	8.0 (6.5–9.5)	7.7 (6.4–9.0)	10.4 (8.9–11.9)
	*Missing*	38								
Unmet health care need because of cost during past 12 months										
	Yes	3319	25.8 (24.2–27.4)	31.2 (27.3–35.0)	36.8 (33.8–39.9)	34.1 (31.4–36.8)	29.3 (27.1–31.5)	32.8 (29.6–36.1)	32.3 (29.6–35.1)	29.1 (27.2–31.1)
	No	10363	74.2 (72.6–75.8)	68.8 (65.0–72.7)	63.2 (60.1–66.2)	65.9 (63.2–68.6)	70.7 (68.5–72.9)	67.2 (63.9–70.4)	67.7 (64.9–70.4)	70.9 (68.9–72.8)
	*Missing*	41								
Routine check-up during past 12 months										
	Yes	10835	79.2 (77.7–80.8)	81.8 (78.5–85.1)	79.1 (76.3–81.9)	80.7 (78.3–83.1)	82.1 (80.0–84.2)	80.8 (77.6–84.0)	81.6 (79.0–84.2)	81.1 (79.3–82.9)
	No	2695	20.8 (19.2–22.3)	18.2 (14.9–21.5)	20.9 (18.1–23.7)	19.3 (16.9–21.7)	17.9 (15.8–20.0)	19.2 (16.0–22.4)	18.4 (15.8–21.0)	18.9 (17.1–20.7)
	*Missing*	193								

**Table 6:** Weighted unadjusted prevalence of health care access by type of disability among 65 years old and above with self-reported COPD, BRFSS 2016–2017.

		All		Hearing	Vision	Cognition	Mobility	Self-care	Independent Living	Any Disability
		N	% (95% CI)	% (95% CI)	% (95% CI)	% (95% CI)	% (95% CI)	% (95% CI)	% (95% CI)	% (95% CI)
**2016**										
Health insurance coverage										
	Yes	19460	98.4 (97.8–98.9)	97.4 (96.5–98.4)	96.9 (95.6–98.3)	97.9 (97.1–98.8)	98.3 (97.8–98.8)	97.1 (95.6–98.6)	97.3 (96.2–98.5)	98.2 (97.8–98.7)
	No	265	1.6 (1.1–2.2)	2.6 (1.6–3.5)	3.1 (1.7–4.4)	2.1 (1.2–2.9)	1.7 (1.2–2.2)	2.9 (1.4–4.4)	2.7 (1.5–3.8)	1.8 (1.3–2.2)
	*Missing*	44								
Usual health care provider										
	Yes	18906	96.2 (95.7–96.8)	96.1 (95.2–97.0)	94.8 (93.3–96.4)	94.1 (92.6–95.7)	96.6 (96.0–97.2)	96.1 (94.8–97.5)	96.4 (95.4–97.4)	96.4 (95.8–96.9)
	No	793	3.8 (3.2–4.3)	3.9 (3.0–4.8)	5.2 (3.6–6.7)	5.9 (4.3–7.4)	3.4 (2.8–4.0)	3.9 (2.5–5.2)	3.6 (2.6–4.6)	3.6 (3.1–4.2)
	*Missing*									
Unmet health care need because of cost during past 12 months										
	Yes	1607	8.2 (7.4–8.9)	9.6 (8.3–11.0)	17.1 (14.1–20.1)	17.1 (14.1–20.1)	11.5 (10.2–12.8)	15.5 (12.6–18.5)	14.8 (12.8–16.8)	10.4 (9.3–11.5)
	No	18109	91.8 (91.1–92.6)	90.4 (89.0–91.7)	82.9 (79.9–85.9)	82.9 (79.9–85.9)	88.5 (87.2–89.8)	84.5 (81.5–87.4)	85.2 (83.2–87.2)	89.6 (88.5–90.7)
	*Missing*	70								
Routine check-up during past 12 months										
	Yes	17604	91.1 (90.2–91.9)	91.5 (89.8–93.3)	90.6 (88.5–92.8)	89.6 (87.4–91.9)	91.1 (90.1–92.2)	89.4 (87.0–91.8)	90.9 (89.5–92.2)	91.2 (90.2–92.2)
	No	1885	8.9 (8.1–9.8)	8.5 (6.7–10.2)	9.4 (7.2–11.5)	10.4 (8.1–12.6)	8.9 (7.8–9.9)	10.6 (8.2–13.0)	9.1 (7.8–10.5)	8.8 (7.8–9.8)
	*Missing*	280								
**2017**										
Health insurance coverage										
	Yes	18102	97.5 (96.5–98.5)	97.1 (96.5–97.7)	94.9 (93.7–96.2)	94.3 (90.7–97.9)	96.7 (95.1–98.3)	92.5 (87.7–97.3)	92.9 (90.5–95.3)	96.7 (95.2–98.1)
	No	266	2.5 (1.5–3.5)	2.9 (2.3–3.5)	5.1 (3.8–6.3)	5.7 (2.1–9.3)	3.3 (1.7–4.9)	7.5 (2.7–12.3)	7.1 (4.7–9.5)	3.3 (1.9–4.8)
	*Missing*	34								
Usual health care provider										
	Yes	17515	95.5 (94.8–96.3)	96.4 (95.6–97.2)	92.4 (91.0–93.9)	93.6 (92.1–95.0)	95.2 (94.3–96.1)	94.4 (93.0–95.8)	93.2 (91.0–95.5)	95.0 (94.0–96.0)
	No	831	4.5 (3.7–5.2)	3.6 (2.8–4.4)	7.6 (6.1–9.0)	6.4 (5.0–7.9)	4.8 (3.9–5.7)	5.6 (4.2–7.0)	6.8 (4.5–9.0)	5.0 (4.0–6.0)
	*Missing*	56								
Unmet health care need because of cost during past 12 months										
	Yes	1482	8.7 (7.8–9.6)	11.1 (9.4–12.7)	16.6 (14.0–19.3)	16.3 (13.5–19.1)	11.7 (10.2–13.2)	18.2 (15.3–21.1)	15.7 (13.2–18.3)	10.8 (9.6–12.1)
	No	16857	91.3 (90.4–92.2)	88.9 (87.3–90.6)	83.4 (80.7–86.0)	83.7 (80.9–86.5)	88.3 (86.8–89.8)	81.8 (78.9–84.7)	84.3 (81.7–86.8)	89.2 (87.9–90.4)
	*Missing*	63								
Routine check-up during past 12 months										
	Yes	16297	90.0 (88.9–91.1)	90.9 (88.3–93.5)	88.2 (85.6–90.9)	87.5 (84.4–90.6)	90.2 (88.7–91.6)	89.3 (86.1–92.5)	89.8 (87.0–92.6)	90.1 (88.9–91.3)
	No	1871	10.0 (8.9–11.1)	9.1 (6.5–11.7)	11.8 (9.1–14.4)	12.5 (9.4–15.6)	9.8 (8.4–11.3)	10.7 (7.5–13.9)	10.2 (7.4–13.0)	9.9 (8.7–11.1)
	*Missing*	234								

## Discussion

To our knowledge, this is the first study to describe the pattern of disability in a national sample of adults with COPD, and to describe healthcare access by disability type in this population. Using self-reported status from the BRFSS in 2016 and 2017, contrary to expectations, we found the prevalence of disabilities to be highest among the 45–64 age group with COPD with exception of hearing. We also found that sex and ethnic disparities existed within each type of disability among individuals with COPD.

It has been well established that a wide range of comorbidities may be seen among individuals with COPD, including metabolic syndrome, sleep apnea, diabetes, depression, osteoporosis, cardiac failure, ischemic heart disease, pulmonary hypertension, lung cancer, muscle wasting.[8] Some of these comorbidities may be directly caused by exposure to tobacco smoke (e.g., ischemic heart disease and lung cancer); others are known consequences of COPD progression (e.g. muscle wasting, pulmonary hypertension and cachexia); and still others have no direct pathophysiological link with COPD but may nonetheless be related with natural progression of the disease (e.g., depression and sleep apnea).[8,11]

COPD is characterized by over-activation of the oxidative metabolism within the airways due to air pollution and/or cigarette smoking.[17] As the often poorly reversible disease progresses, individuals experience loss of lean muscle mass and physical activity becomes increasingly difficult.[18,19] Therefore, it is plausible that disabilities related to strength and physicality such as mobility, self-care, and independent living could be connected to the severity of the disease. However, the association between the progression from COPD and disabilities has not been extensively studied, more so among adults 45–64 years old. The evidence put forth with this study surely warrants further research to fully grasp the extent of COPD-related disability burden. Moreover, understanding the temporality between the various disabilities and the progression of severe COPD would be of particular importance as both the prevalence of COPD and disabilities are rising.[12,18]

The available evidence has shown that the most common chronic conditions causing disabilities among adults are rheumatoid arthritis, spine problems, and cardiovascular disease.[20] However, these conditions increase in prevalence as the population ages, and do not fully explain the highest rates seen in the middle age group in the current study. In the general adult population, rates of disability increase with each increasing age group and are highest for cognitive limitations in the middle age group.[12,21] The reasons for the observed age-specific differences in disabilities related to hearing, vision, cognition, mobility, self-care, and independent living among adults with COPD are not completely understood. These types of disabilities may affect the ability to successfully manage COPD, from reading prescription or medication labels, to transportation for primary care appointments. Currently there are public resources available for Medicare enrollees, those over the age of 65, that support these needs.[22] Our study findings highlighted the existence of an unmet need for support among the 45–64 year old, not necessarily encompassed in eligibility and outreach of assistance to meet healthcare needs. A recently published analysis of national trends in disability from 2002 to 2016 also found an increasing prevalence among the same age group, specifically a 23–25% increase during the time frame, explained theoretically by increases in various chronic diseases and declines in economic conditions.[23] While our claims were similar regarding the burden of chronic conditions among the 45–64 age group, the aforementioned project did not, however, consider other age groups. It is therefore possible that similar trends could be observed across other studies.

The current study observed a higher proportion of women in the study sample with COPD, 57.4%, a finding consistent with higher prevalence of COPD among women nationally while the overall national survey sample was 51% women[1,24]. While previously underdiagnosed in women, recent studies have found that there are gender-specific differences in susceptibly to risk factors for COPD such as smoking and response to treatment.[25] Our findings of disability among women with COPD also align with studies of gender differences in the prevalence of disability in the general population.[12] The previous study found that the rates were higher among women of all age groups except for hearing and self-care. This finding was consistent among females with COPD surveyed. Although the prevalence of COPD is overall higher among women in the US, the pattern of disability does not differ from women without COPD. Yet, women with COPD experience a higher risk of exacerbations and more pronounced symptoms then men with COPD.[26] Further research is needed to fully explain this finding.

Differences in healthcare access among adults with COPD among the four measured were found. Regardless of age group and type of disability, adults with COPD were found to have higher rates of health insurance coverage, primary care provider, and routine yearly checkup than the general population.[12] However, the middle age-group, adults that typically are still in the workforce were less likely to have health insurance coverage than the older age group, usually covered with a version of public healthcare insurance.[27]

Rates of cost deterrence to meet healthcare needs were higher among adults with COPD compared to the general population.[12] This finding is not surprising as the impact of the cost burden of chronic diseases and disabilities can place on an individual’s ability to respond to other healthcare needs has long been recognized.[28,29] It further emphasizes that as middle-aged adults are diagnosed with COPD and see higher rates of disabilities, healthcare cost issues are now happening in a group typically still in the workforce, and will likely continue throughout the individual’s lifetime.

Ethnic differences in hearing disabilities among people with COPD were observed whereby among Black/African American with COPD, prevalence rates of hearing disabilities were lowest but among the highest for other disabilities. Previous research among older adults has shown that African Americans and Spanish-speaking Hispanics 65 and older are more likely to develop a disability.[30] However, in the current study, adults without further age restrictions, lower rates of hearing disabilities among African American surveyed could be explained by the age distribution of the sampled population, the weighted majority being less than 65 years old. It is difficult to explain this finding in the context of the study as there is very limited contemporaneous evidence relating to racial disparities in hearing disability precisely and related ramifications to sampling representation in national surveys. It would be worthwhile to further investigate the epidemiological characteristics of this disparity.

### Strengths and limitations

While our analysis was rigorous, these findings have a few limitations. The assessment of the prevalence of disability, the COPD status, and healthcare access are all based on self-reports made during a telephone survey. These answers are also subject to recall bias, as well as, social desirability bias from the telephone surveying methodology. The inability to confirm COPD status should be taken in account. Nonetheless, results from the BRFSS survey methodologies have been cross-validated with other population-wide data sources.[31] Additionally, in the context of a national telephone survey, it is difficult to completely explain all observations as it is likely that marginalized people with COPD and/or disabilities may not be accurately represented in the sample.[32,33] These individuals may be less likely to have accessed the healthcare system in order to be informed of their COPD status and were therefore not accounted in this study While these limitations may skew the results of the study, the confidence in our results is aided by the strong record of national survey sampling methodology, in addition to a well-developed questionnaire.

## Conclusions

The study of the prevalence of disability among adults with COPD nationally adds to the evidence that disparities exist, in terms of age and gender by type of disabilities, and healthcare access among adults with COPD and disabilities. While the multi-level causes of these disparities should be investigated further, programs supporting those with COPD should take in account capacities that may impact the success of long-term care plans.
